# Plantar calcaneal spurs in older people: longitudinal traction or vertical compression?

**DOI:** 10.1186/1757-1146-1-7

**Published:** 2008-08-11

**Authors:** Hylton B Menz, Gerard V Zammit, Karl B Landorf, Shannon E Munteanu

**Affiliations:** 1 Musculoskeletal Research Centre, Faculty of Health Sciences, La Trobe University, Bundoora, Victoria, 3086, Australia; 2 Department of Podiatry, Faculty of Health Sciences, La Trobe University, Bundoora, Victoria, 3086, Australia

## Abstract

**Background:**

Plantar calcaneal spurs are common, however their pathophysiology is poorly understood. This study aimed to evaluate the prevalence and correlates of plantar calcaneal spurs in a large sample of older people.

**Methods:**

Weightbearing lateral foot radiographs of 216 people (140 women and 76 men) aged 62 to 94 years (mean age 75.9, SD 6.6) were examined for plantar calcaneal and Achilles tendon spurs. Associations between the presence of spurs and sex, body mass index, radiographic measures of foot posture, self-reported co-morbidities and current or previous heel pain were then explored.

**Results:**

Of the 216 participants, 119 (55%) had at least one plantar calcaneal spur and 103 (48%) had at least one Achilles tendon spur. Those with plantar calcaneal spurs were more likely to have Achilles tendon spurs (odds ratio [OR] = 2.0, 95% confidence interval [CI] 1.2 to 3.5). Prevalence of spurs did not differ according to sex. Participants with plantar calcaneal spurs were more likely to be obese (OR = 7.9, 95% CI 3.6 to 17.0), report osteoarthritis (OR = 2.6, 95% CI 1.6 to 4.8) and have current or previous heel pain (OR = 4.6, 95% CI 2.3 to 9.4). No relationship was found between the presence of calcaneal spurs and radiographic measures of foot posture.

**Conclusion:**

Calcaneal spurs are common in older men and women and are related to obesity, osteoarthritis and current or previous heel pain, but are unrelated to radiographic measurements of foot posture. These findings support the theory that plantar calcaneal spurs may be an adaptive response to vertical compression of the heel rather than longitudinal traction at the calcaneal enthesis.

## Background

Osseous spurring of the plantar aspect of the calcaneus was first documented in 1900 by the German physician Plettner, who coined the term *Kalkaneussporn *(calcaneal spur) [1]. Although initially considered to be an abnormal finding inextricably linked to heel pain, more recent studies have reported that between 11 and 16% of the general population have radiographic evidence of calcaneal spurs [2-8]. Nevertheless, it does appear that calcaneal spurs are over-represented in particular subgroups, including older people [3,5,8,9], females [4,5,7], people with osteoarthritis [3,9] and people with previous or current heel pain [2,4,6,10,11]. The association between calcaneal spurs and heel pain has led to the development of several interventions directly targeted at the spur, including surgical excision [12], extracorporeal shockwave therapy [13] and radiation therapy [14].

The pathophysiology of calcaneal spurs is poorly understood. The traditional explanation, which could be termed the *longitudinal traction hypothesis*, suggests that repetitive traction of the insertion of the plantar fascia into the calcaneus leads to inflammation and reactive ossification of the enthesis [15]. Evidence to support this hypothesis can be derived from studies which have shown that plantar fascial tension increases with lowering of the medial longitudinal arch [16], and that people with heel pain are more likely to be flatfooted [6,17]. However, the validity of this hypothesis has also been questioned by studies which have shown that: (i) most spurs are located deep to the plantar fascia (typically in the flexor digitorum brevis, quadratus plantae and abductor hallucis muscle insertions [18-22], but also within fibrocartilage and loose connective tissue [23]); (ii) histological analysis of surgically excised plantar fascia does not reveal signs of inflammation [24]; (iii) the bony trabeculae of spurs are not aligned in the direction of soft tissue traction [23]; and (iv) excised spurs can reform after surgical release of the plantar fascia [25].

An alternative explanation proposed by Kumai and Benjamin [26], which could be termed the *vertical compression hypothesis*, argues that calcaneal spurs develop in response to repetitive compression rather than traction. Specifically, they suggest that calcaneal spurs are fibrocartilagenous outgrowths which form in response to calcaneal stress fractures, in an attempt to protect the calcaneus against microcracks [26]. Such an explanation is consistent with studies which have found that calcaneal spurs are more common in those who are overweight [27], and in those who have decreased elasticity of the plantar heel fat pad [28], such as older people [29]. Furthermore, a recent histological study has indicated that the bony trabeculae of spurs are vertically oriented, suggesting that the stresses responsible for spur formation may be the result of vertical loading [23].

No studies have specifically evaluated the prevalence and correlates of calcaneal spurs in older people. This is despite the fact that heel pain is common in this age-group [30], as are several factors known to be associated with calcaneal spurs and heel pain, such as osteoarthritis [31], obesity [32] and flatfoot [33]. Therefore, the aim of this study was to explore the associations between calcaneal spurs, heel pain, obesity, foot posture and osteoarthritis in a sample of older people. In doing so, our objective was to provide further insights into the aetiology of calcaneal spurs, which may have implications for the management of heel pain in this population.

## Methods

### Participants

The sample comprised 216 people (76 men and 140 women) aged between 62 and 94 years (mean 75.9, SD 6.6) who were taking part in a larger study of the effect of osteoarthritis on balance and falls. Participants were recruited from two sources: a retirement village (n = 95) and a university health sciences clinic (n = 121). The exclusion criteria were a history of Parkinson's disease, inability to walk household distances without the use of a walking aid, or a score of less than 7 on the Short Portable Mental Status Questionnaire [34].

Major medical conditions and presence of pain were determined through a structured interview. Reporting of major medical conditions involved a simple checklist of conditions, with the question "Do you have/have you ever had the following conditions?". Those who reported having osteoarthritis were then requested to indicate the location of their osteoarthritis from a checklist including hands, spine, hips, knees or feet. In relation to pain, participants were asked "Do you have/have you ever had the following symptoms?" followed by a checklist including back/neck pain, hip pain, hand/wrist pain, knee/leg pain and foot pain. Those who reported foot pain were then requested to indicate the location of their foot pain on a diagram showing dorsal, plantar, medial and lateral images of the foot. Body mass index (BMI) was documented as weight (in kilograms)/height (in metres)^2^, and obesity was defined as a BMI > 30 kg/m^2^. The Human Studies Ethics Committee at La Trobe University and the Radiation Advisory Committee of the Victorian Department of Human Services approved the study, and written informed consent was obtained from all participants.

### Radiographic procedure and documentation of spurs

**Figure 1:**
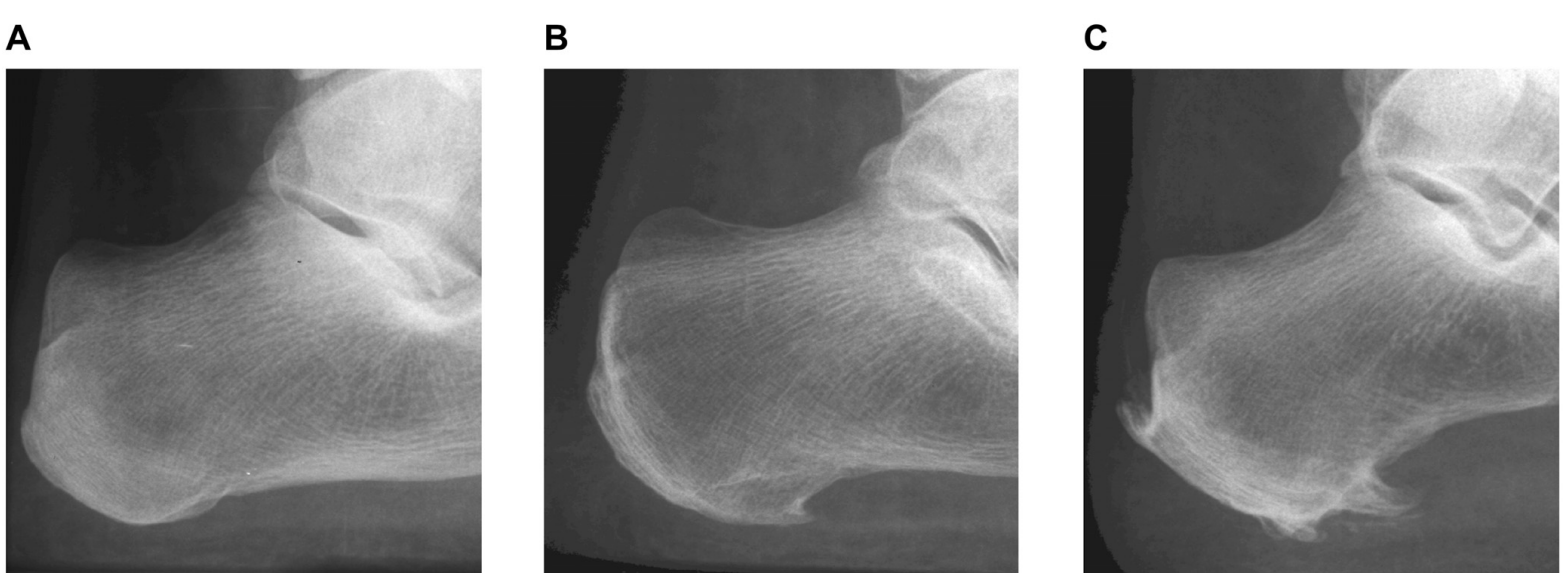
**Examples of x-rays obtained in the study**. A: no plantar calcaneal or Achilles tendon spur, B: plantar calcaneal spur only, C: plantar calcaneal spur and Achilles tendon spur.

Weightbearing lateral radiographic projections were obtained from both feet with the participant standing in a relaxed bipedal stance position. All x-rays were taken by the same medical imaging department using a Shimadzu UD150LRII 50 kw/30 kHz Generator and 0.6/1.2 P18DE-80S high speed x-ray tube from a ceiling suspended tube mount. AGFA MD40 CR digital phosphor plates in a 24 cm × 30 cm cassette were used. The tube was angled 90 degrees and centered at the base of the third metatarsal. The film focus distance was set at 100 cm. All radiographs were initially screened for plantar calcaneal and Achilles tendon spurs by one of the authors (GVZ), and were classified as (i) no spur evident (ii) definite spur, or (iii) possible spur. The radiographs were then re-examined by three authors (GVZ, HBM, KBL), who reached a final determination (i.e.: spur present or absent) by consensus. Due to their irregular shape and orientation, no attempt was made to directly measure the length of the spurs. Furthermore, the clarity of the x-rays did not allow for any delineation between spurs located in the plantar fascia and those located in the intrinsic musculature. Examples of the x-rays obtained in the study are shown in Figure 1.

### Radiographic foot posture measurement

**Figure 2:**
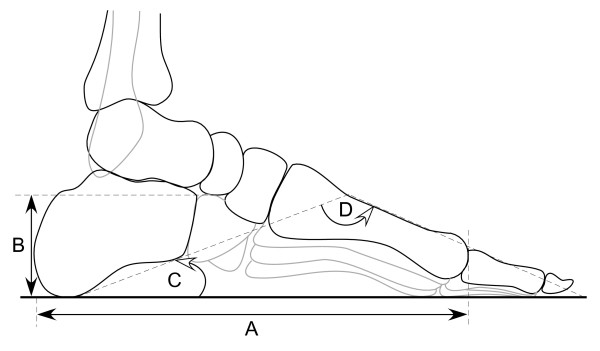
**Foot posture measurements obtained from lateral projection**. A = truncated foot length, B = navicular height, C = calcaneal inclination angle, D = calcaneal-first metatarsal angle.

Each radiograph was placed on a horizontally positioned viewing box and covered with overhead transparency film. Three foot posture measurements were then obtained (Figure 2). Navicular height was measured as the distance between the supporting surface and the inferior border of the navicular bone, and was normalised for foot size by dividing it by the distance between the posterior aspect of the calcaneus and the most distal border of the first metatarsal head [35]. Calcaneal inclination angle was defined as the angle between the tangent of the inferior surface of the calcaneus and the supporting surface, with a lesser score indicating a flatter foot [36]. Calcaneal-first metatarsal angle was defined as the angle subtended by the tangent to the inferior surface of the calcaneus and a line drawn along the dorsum of the midshaft of the first metatarsal. A greater calcaneal-first metatarsal angle indicates a flatter foot [36]. The high reliability of these measurements has been documented previously (intraclass correlation coefficients ≥ 0.98) [37].

### Statistical analysis

All statistical tests were conducted using SPSS Release 14.0 for Windows (SPSS Inc, Chicago, IL). Comparisons between participants with and without calcaneal spurs were undertaken using independent samples *t*-tests for continuously scored variables, and chi-square tests and odds ratios (OR) for dichotomous variables. Foot posture measurements were analysed both as continuous variables and as dichotomous variables by transforming them into quartiles. A logistic regression analysis was then undertaken to determine which variables were most strongly associated with the presence of calcaneal spurs, after adjusting for age, sex, and the presence of Achilles tendon spurs. For all statistical tests, the level of significance was set at *p *< 0.05.

## Results

Of the 216 participants, 119 (55%) had at least one plantar calcaneal spur and 103 (48%) had at least one Achilles tendon spur. Comparisons between those with and without plantar calcaneal spurs are shown in Table 1. Prevalence of spurs did not differ according to sex. However, participants with plantar calcaneal spurs were significantly more likely to be obese (OR = 7.9, 95% CI 3.6 to 17.0), report osteoarthritis in at least one body region (OR = 2.6, 95% CI 1.6 to 4.8) and have current or previous heel pain (OR = 4.6, 95% CI 2.3 to 9.4). Achilles tendon spurs were also significantly more common in those with plantar calcaneal spurs (OR = 2.0, 95% CI 1.2 to 3.5). There were no significant differences between the groups in relation to the mean values for the three radiographic measurements of foot posture. Similarly, there were no significant differences in the frequency of calcaneal spurs across the quartile categories for each of the foot posture measurements.

**Table 1:** Characteristics of participants with and without calcaneal spurs.

	Calcaneal spur absent (n = 97)	Calcaneal spur present (n = 119)
Age (years)	76.2 (7.0)	75.8 (6.2)
Female – n (%)	64 (66)	76 (64)
Obese – n (%)	9 (9)	53 (45)*
Major medical conditions – n (%)		
Osteoarthritis	57 (59)	94 (80)*
Rheumatoid arthritis	5 (5)	2 (2)
Diabetes mellitus	15 (16)	17 (14)
Stroke	6 (6)	3 (3)
Peripheral vascular disease	12 (12)	15 (13)
Hypertension	54 (56)	76 (64)
Current or previous heel pain – n (%)	12 (12)	47 (40)*
Achilles tendon spur – n (%)	37 (38)	66 (56)*
Radiographic foot posture measures		
Calcaneal inclination angle (°)	21.2 (5.1)	20.4 (6.1)
Calcaneal-first metatarsal angle (°)	132.5 (7.5)	133.8 (8.9)
Navicular height (mm)	30.2 (5.2)	30.9 (6.3)

* significant difference for χ^2 ^test, *p *< 0.01

Results of the logistic regression analysis are shown in Table 2. After adjustment for age, sex, and presence of Achilles spurs, three variables were shown to be significantly and independently associated with calcaneal spurs: obesity, current or previous heel pain, and osteoarthritis.

**Table 2:** Results of logistic regression, adjusted for age, sex, and presence of Achilles spurs.

Predictor variable	Odds ratio (95% CI)	*p *value
Obese	6.9 (3.0 to 15.8)	< 0.001
Current or previous heel pain	3.9 (1.8 to 8.4)	0.001
Osteoarthritis	2.3 (1.2 to 4.6)	0.017

## Discussion

The aim of this study was to evaluate the prevalence and correlates of plantar calcaneal spurs in a large sample of older people. We found that 55% of the sample had at least one calcaneal spur, which is considerably higher than the 11 to 16% range that has been previously reported in young to middle aged populations [2-8]. Our results are similar to those of Bassiouni [3], who reported a 72% prevalence of calcaneal spurs in rheumatology patients aged "above 61 years", and Banadda et al [5], who reported a 50% prevalence of spurs in Zimbabwean hospital patients aged "over 51 years". Although prospective studies would be required to confirm whether the prevalence of spurs increases with age, the Banadda et al [5] study demonstrated a linear increase in the prevalence of calcaneal spurs across five age-bands ranging from 11 to 20 years to over 51 years.

The strongest association with calcaneal spurs was obesity, with 45% of participants classified as obese having spurs, compared to only 9% of those who were not obese. Although obesity is a well-recognised risk factor for heel pain [38], to our knowledge only one previous study, conducted in military recruits, has reported a positive association between increased bodyweight and calcaneal spurs [27]. This association is consistent with the vertical compression hypothesis of spur formation, as several studies have shown that vertical heel pressure during gait is strongly associated with bodyweight [39,40]. Excess body mass may accelerate the degenerative processes occurring in the plantar heel region, particularly in the presence of age-related stiffening of the plantar heel pad [29]. However, it is also possible that obesity results in greater flattening of the medial longitudinal arch, which then creates additional traction on the plantar fascial insertion and subsequent spur development.

Plantar calcaneal spurs were also significantly associated with osteoarthritis, which is in agreement with two previous studies [3,9]. However, no association was found between calcaneal spurs and other major medical conditions (rheumatoid arthritis, diabetes mellitus, stroke, peripheral vascular disease or hypertension). Although the sample size in our study was probably too small to detect significant associations with these conditions, previous studies with larger samples have found no difference in the prevalence of spurs in people with or without diabetes mellitus [41] and only a slightly higher prevalence in people with rheumatoid arthritis (22% compared to 16% of controls) [3]. The association between plantar calcaneal spurs and osteoarthritis is also compatible with the vertical compression hypothesis [26], as degenerative changes in enthesis fibrocartilage and the formation of subchondral sclerosis, processes that are thought to be responsible for spur formation, are likely to be augmented in the presence of osteoarthritis. Indeed, a positive association between spur formation and osteophytes has been reported [42].

Consistent with several previous studies [2,4,6,10,11], participants with plantar calcaneal spurs were more likely to have current or previous heel pain, although a substantial proportion of those with spurs (61%) were asymptomatic. Clearly, the presence of a plantar calcaneal spur does not always lead to the development of heel pain. Why some spurs are associated with symptoms while others are not is yet to be adequately investigated, but possible explanations include the size of the spur (i.e. very large spurs may be more likely to be symptomatic [11]), the presence of concurrent fat pad abnormalities leading to increased shock transmission to the spur [43], entrapment of the nerve to abductor digiti minimi caused by the spur [44], and fracture of the spur itself [22]. It is also possible that extrinsic factors, such as footwear, occupational environment and level of physical activity may play a role in determining whether people with plantar calcaneal spurs develop symptoms. Each of these suggestions warrants further investigation.

Foot posture, determined using three radiographic measurements, was not associated with calcaneal spurs, irrespective of whether these measures were expressed as continuous variables or divided into quartile categories. This finding is incompatible with the longitudinal traction hypothesis, which suggests that spurs form in response to repetitive traction of the plantar fascial insertion, a process that is thought to be exacerbated in people with pronated or flat feet [15]. Given the wide range of foot postures evident in our sample (from very highly arched to very flat), we would have expected at least some indication of a trend towards flatter feet if this hypothesis was correct. Although we acknowledge that the use of kinematic measures to assess dynamic arch flattening during gait may provide more useful insights into the potential role of longitudinal traction in the pathogenesis of spurs, such a mechanism is inconsistent with the histological evidence of vertically-aligned bony trabeculae found in spurs [23]. On the basis of these findings, we cautiously suggest that while flat feet may indeed be associated with heel pain (and that the mechanism may be related to longitudinal traction of the plantar fascia), the formation of calcaneal spurs may be more closely associated with compression than traction.

These findings need to be interpreted in the context of several limitations in the study design. Firstly, the sample was not randomly selected, so the prevalence of calcaneal spurs reported here may not be generalisable to the broader community. Secondly, the presence of major medical conditions was determined by self-report. Although self-reported medical history in older people has been shown to be accurate for most conditions, it is generally less accurate for osteoarthritis, with a tendency for women to over-report and men to under-report [45]. Thirdly, heel pain was documented as being present or absent, and no attempt was made to determine the underlying cause of the pain. As such, it is possible that cases of heel pain with a non-mechanical aetiology may have been included. Finally, as with all cross-sectional studies, causal relationships cannot be inferred from the data. While the associations described here are physiologically plausible, further research is required to confirm causation.

## Conclusion

Plantar calcaneal spurs are highly prevalent in older people, and are associated with obesity, osteoarthritis and current or previous heel pain, but are unrelated to foot posture. In conjunction with previous reports in the literature, these findings support the theory that plantar calcaneal spurs may primarily be an adaptive response to vertical compression of the heel rather than longitudinal traction at the calcaneal enthesis, which may have implications for the management of chronic heel pain in older people.

## Competing interests

HBM and KBL are Editor-in-Chief and Deputy Editor-in-Chief, respectively, of the *Journal of Foot and Ankle Research*. It is journal policy that editors are removed from the peer review and editorial decision making processes for papers they have co-authored.

## Authors' contributions

HBM conceived the study, analysed and interpreted the data, and drafted the manuscript. GVZ, KBL and SEM assisted with data collection and interpretation. All authors read and approved the final version of the manuscript.
